# Associations of Lifestyle Factors With Cognition in Community-Dwelling Adults Aged 50 and Older: A Longitudinal Cohort Study

**DOI:** 10.3389/fnagi.2020.601487

**Published:** 2020-11-09

**Authors:** Zhezhou Huang, Yanfei Guo, Ye Ruan, Shuangyuan Sun, Tao Lin, Jinghong Ye, Jun Li, Lihua He, Sen Wang, Yan Shi, Fan Wu

**Affiliations:** ^1^School of Public Health, Fudan University, Shanghai, China; ^2^Shanghai Municipal Center for Disease Control and Prevention, Shanghai, China; ^3^Pudong New District Center for Disease Control and Prevention, Shanghai, China; ^4^Hongkou District Center for Disease Control and Prevention, Shanghai, China; ^5^Minhang District Center for Disease Control and Prevention, Shanghai, China; ^6^Huangpu District Center for Disease Control and Prevention, Shanghai, China; ^7^Qingpu District Center for Disease Control and Prevention, Shanghai, China

**Keywords:** aging, cognitive decline, lifestyle, vegetable, fruit, physical activity, obesity, sedentary time

## Abstract

In the absence of an effective treatment to alter the progressive course of cognitive decline and dementia, identification of modifiable risk factors that could promote healthy cognitive aging has become a public health research priority. This study seeks to comprehensively determine the contemporaneous associations of a broad spectrum of time-varying modifiable lifestyle factors with age-related cognitive decline in a large population-based cohort of older adults. A total of 5,711 subjects aged 50 and older from the WHO Study on global AGEing and adult health (SAGE) in Shanghai were studied. Repeated measures of lifestyle factors and cognitive performance were conducted in 2009–2010 and 2014–2015. Linear random slope models were used to evaluate the contemporaneous associations between time-varying lifestyle factors and cognitive performance. Person-mean centering method was used to disaggregate the between- and within-person effects in the time-varying lifestyle factors in the random slope models. We found that higher vegetable and fruit consumption, as well as higher level of physical activity were positively associated with all cognitive domains. Body mass index (BMI) was negatively associated with all cognitive domains, whereas waist-to-hip ratio (WHR) was negatively associated with verbal fluency score only. Sedentary time was negatively associated with digit span score but positively associated with verbal fluency score. The between-person effects seem to be more dominant than within-person effects. Overall, our findings suggest better management of multiple lifestyle factors may protect against cognitive decline in later life. Higher vegetable and fruit consumption and physical activity are protective, whereas obesity is detrimental to cognitive decline in older adults. This study underpins the development of multi-domain lifestyle recommendations to promote healthy cognitive aging.

## Introduction

Aging is accompanied by cognitive decline which is evident from as early as age 45 (Singh-Manoux et al., 2012). Age-related cognitive decline manifests itself as some diminishment of many core cognitive abilities, including memory, attention, processing speed, and executive function (Park and Bischof, 2013). The different levels of cognitive decline range from mild cognitive impairment to dementia (Langa and Levine, 2014). Dementia is one of the major causes of disability and dependency among older people worldwide. Dementia currently affects approximately 50 million people worldwide, with the majority (63%) living in low- and middle-income countries (WHO, 2020b). The total number of people with dementia is projected to reach 82 million by 2030 and 152 million by 2050 (WHO, 2020b). The number of patients with dementia in China accounts for approximately a quarter of the entire population with dementia worldwide, with a prevalence of 5.30 and 12.7% for dementia and mild cognitive impairment, respectively, in people aged 60 years and older, and an incidence of 12.14 and 21.7 per 1,000 person-years for dementia and mild cognitive impairment, respectively, in people aged 65 years and older (Jia et al., 2020). Therefore, there is an urgent need to slow down cognitive decline and halt the progression to dementia.

Since no effective pharmacological treatment is currently available to cure dementia, a greater emphasis has been placed on identification of potentially modifiable risk factors that could delay or prevent cognitive decline. Findings from prior research have provided promising results linking several modifiable risk factors to cognitive impairment and dementia. It is estimated that around a third of Alzheimer’s diseases cases worldwide might be attributable to seven potentially modifiable risk factors (diabetes, midlife hypertension, midlife obesity, physical inactivity, depression, smoking, and low educational attainment) (Norton et al., 2014). Over the past decades, emerging evidence suggests that a “poor” lifestyle such as obesity, physical inactivity, unhealthy diet, smoking, and heavy alcohol consumption are associated with faster cognitive decline in old age (Wajman et al., 2018). Such evidence highlights the potential of promoting healthy lifestyles to preserve cognition in normal aging.

To date, the majority of previous studies investigating the relationship between lifestyle factors and cognition have used cross-sectional designs (Arenaza-Urquijo et al., 2015). However, age-related cognitive decline is highly variable across individuals (Nyberg et al., 2012). The cross-sectional design cannot estimate the effects of lifestyle factors on cognitive decline over time at an intra-individual level. Evidence from longitudinal studies, cross-lagged panel analyses and intervention trials has accumulated during the past few decades. The cross-lagged panel model excels in taking temporal orders into account and thus sheds light on the temporal sequence and causal direction of the associations between lifestyle factors and cognition (Farina et al., 2016; Ihle et al., 2019; Zhao et al., 2020). Despite this, most studies focus on only one or two lifestyle factors (Lövdén et al., 2013; Kivipelto et al., 2018). Cognitive decline are multifactorial in nature (Kivipelto et al., 2018). Risk factors and protective factors often co-occur and interact across a person’s lifespan, and the result of which determines the overall risk of cognitive decline (Kivipelto et al., 2018). Previous single-domain studies did not consider the possible interactive effects between different lifestyle factors, and have yielded mixed results (Kivipelto et al., 2018). Consequently, studies that target multi-domain lifestyle factors simultaneously are needed to provide overall and integrated recommendations for how to delay cognitive decline in the general population. Furthermore, while studying the effects of lifestyle behavior on health, most epidemiological studies assume relatively stable behavioral patterns. However, lifestyle behavior is subject to fluctuations over time, and these fluctuations are not taken into account when evaluating the effects of lifestyle behavior on future health status (Mulder et al., 1998). As a consequence, the effects of lifestyle behavior on health are based on one single measurement. Research into temporally parallel changes in lifestyle behavior and changes in cognition at an intra-individual level is rather scarce, although this kind of study will provide the best insight into the contemporaneous associations between lifestyle behavior and cognitive functioning. Lastly, the majority of the epidemiological studies on lifestyle factors and cognition have only been carried out in high-income countries (Kivipelto et al., 2018). There is little published data from low-income and middle-income countries, which are facing the greatest burden of dementia.

To fill the above mentioned gap, this study seeks to explore the contemporaneous associations between a broad spectrum of time-varying modifiable lifestyle factors and cognition, as well as potential interactive effects between different lifestyle factors, in normal aging in a large longitudinal population-based cohort of Chinese adults aged 50 and older.

## Materials and Methods

### Study Population

Participants were drawn from a large ongoing population-based cohort study, the WHO Study on global AGEing and adult health (SAGE) in Shanghai. Detailed descriptions of SAGE have been previously described (Kowal et al., 2012). Briefly, SAGE is a longitudinal study collecting data on adults aged 50 and older from nationally representative samples in China, Ghana, India, Mexico, Russian Federation and South Africa. We enlarged the sample size of SAGE in Shanghai, China to obtain a sub-state representative sample using the same multistage clustered sampling method and survey instrument. At baseline (Wave 1) between 2009 and 2010, 8,629 community dwellers aged 50 and older were recruited from five districts of Shanghai, China. Of subjects recruited, 74 who used proxy, 418 who had a history of stroke, and 137 who did not complete cognitive tests at Wave 1 were excluded; leaving 8,000 participants eligible for this study. Among the eligible participants, 5,711 completed cognitive tests at Wave 2 between 2014 and 2015, and were included in this study. This study was approved by the Shanghai Center for Disease Control and Prevention Ethical Review Committee. All participants provided informed written consent.

### Lifestyle Factors

Lifestyle factors included vegetable and fruit intake (100 g/day), physical activity, sedentary time (hours/day), body mass index (BMI) and waist-to-hip ratio (WHR). All lifestyle factors were assessed at both Wave 1 and Wave 2. Participants were asked to indicate the number of servings of fruits and vegetables they consume on a typical day. One serving of fruits and vegetables is equivalent to 50 g in Wave 1 questionnaire and 80 g in Wave 2 questionnaire. In order to unify the measuring units and make them more comparable to other studies, we then converted the self-reported number of servings of fruits and vegetables to 100 g/day as an indicator for general fruit and vegetable intake. Physical activity level was measured based on responses to questions drawn from the Global Physical Activity Questionnaire (WHO, 2020a). Three categories of physical activity (low, moderate, and high physical activity) were calculated from these questionnaire items, based on reported time spent on moderate or vigorous activities during work, recreational/leisure time, and transportation (International Physical Activity Questionnaire [IPAQ], 2005). Trained doctors measured participants’ weight, height and waist circumference. BMI was calculated by dividing the body weight (in kilograms) by the height (in meters) squared (kg/m^2^). WHR was calculated as waist measurement divided by hip measurement. Sedentary time (hours/day) was measured based on the time spend sitting or reclining on a typical day.

### Cognitive Function

Cognitive function was assessed through the following cognitive tests: (1) immediate verbal recall to assess learning capacity and memory storage (Morris et al., 1989); (2) delayed verbal recall to assess memory retrieval (Morris et al., 1989); (3) digit span forward and backward to assess concentration, attention and immediate memory (The Psychological Corporation, 2002); and (4) verbal fluency test to assess ability to retrieve semantic memory information (Morris et al., 1989). For the immediate verbal recall test, interviewers read a list of 10 words aloud and asked participants to immediately recall as many words as possible in 1 min. This test was performed three times. The final score was the average of three tests. After 10 min, delayed verbal recall test was performed by asking participants to remember the list of words without interviewers repeating the list. Digit span test asked participants to repeat increasingly longer series of numbers; the total score was recorded as the longest digit span repeated without error. This test was then performed with participants repeating new sets of increasingly longer digit spans in reverse. Verbal fluency test consisted of naming as many animals as possible in 1 min; the total score was correct responses minus errors.

### Covariates

Covariates included age, sex, education, smoking (non-smoker and smoker), alcohol drinking (non-drinker and drinker), and self-reported common chronic health conditions including arthritis, angina, diabetes, chronic lung disease, asthma, hypertension, depression, and cataract.

### Statistical Analyses

All analyses were performed using Stata 16.0. Statistics were presented as mean (SD) or n (%) where appropriate. *T*-test for paired samples was used to test the differences between cognitive scores at Wave 1 and Wave 2. Since repeated measurements of cognitive performance and lifestyle factors are two-level hierarchical data, with measurement occasions at level 1 and individuals at level 2, we analyzed the associations of cognitive performance with lifestyle factors using linear random slope models with maximum-likelihood estimates. Specifically, regression models were fitted to each individual’s longitudinal cognitive change, resulting in an average model for the sample (fixed effects) plus individual deviations from the average model (random effects). Subject ID was included as a random effect. Age as the time scale, common chronic health conditions and lifestyle factors were all time-varying variables. As for random effects we included subject ID to account for repeated data of the same individuals. Coefficient of determination (*R*^2^) was presented to indicate the proportion of level 1 and level 2 variances explained by the covariates.

To disaggregate the between- and within-person effects in the time-varying lifestyle factors in random slope models, we used person-mean centering method which involves recalculating a single time varying lifestyle factor into separate between- and within-person predictor variables: at level 1, the person-mean centered time-varying lifestyle factor serves as a within-person predictor, representing the amount by which a person deviates from his or her own average at each time point (within-person effect); at level 2, the person means serve as a between-person predictor, representing each person’s average, pooling over all time points (between-person effect) (Howard, 2015).

We first evaluated the associations of a single lifestyle factor with cognitive scores separately, and then included all lifestyle factors in the same model (full model). To test for potential interactions between different lifestyle factors, as well as interactions between lifestyle factor and demographic factors (age and sex), we then included interaction terms in the full model. Lastly, to allow for a time lag between lifestyle factors and cognitive functioning, we also examined the associations between baseline lifestyle factors and longitudinal cognitive scores.

## Results

Of 5,711 subjects who finished the cognitive tests at both Wave 1 and Wave 2 with a mean interval of 4.89 years, 2,640 (46.28%) were men and 3,064 (53.72%) were women. The subjects ranged in age from 50 to 95, with a mean age of 62.29 at baseline. As shown in Table 1, at baseline, our participants had a mean vegetable and fruit intake of 369 g/day, mean BMI of 24.71 kg/m^2^, mean WHR of 0.89, mean sedentary time of 4.10 h/day, 59.66% had only a low level of physical activity. Compared with participants who dropped out from the second cognitive assessment (*n* = 2,289), those who completed both cognitive assessments (*n* = 5,711) were 2.12 years younger (*p* < 0.01), more likely to be smokers (*p* < 0.01) and drinkers (*p* = 0.01), more likely to have arthritis (*p* < 0.01) but less likely to have cataract (*p* < 0.01) and diabetes (*p* = 0.03), had a lower education attainment (*p* < 0.01), a higher BMI (*p* < 0.01), shorter daily sedentary time (*p* < 0.01), a higher immediate verbal recall score (*p* < 0.01) and digit span score (*p* < 0.01), and a lower verbal fluency score (*p* < 0.01).

**TABLE 1:** Comparisons of baseline characteristics between participants completed both cognitive assessments and those who dropped out from the second cognitive assessment.

	**Participants completed the first cognitive assessment**		**Participants completed both cognitive assessments**		**Participants dropped out from the second cognitive assessment**		***P*^c^**
		
	**(*n* = 8,000)**		**(*n* = 5,711)**		**(*n* = 2,289)**		
	**Mean/*N*^a^**	***SD*/%^b^**	**Mean/*N*^a^**	***SD*/%^b^**	**Mean/*N*^a^**	***SD*/%^b^**	
Age	62.90	9.53	62.29	8.84	64.41	10.91	< 0.01
Sex							0.42
Men	3,691	46.57	2,640	46.28	1,051	47.30	
Women	4,235	53.43	3,064	53.72	1,171	52.70	
Education attainment							< 0.01
Lower than primary school	1,344	19.66	1,035	21.28	309	15.67	
Primary school	1,378	20.16	1,064	21.88	314	15.92	
Middle school	2,221	32.49	1,534	31.54	687	34.84	
High school	1,386	20.28	906	18.63	480	24.34	
College or higher	507	7.42	325	6.68	182	9.23	
Yearly income (¥)	20233.73	95276.82	19142.80	90898.20	22993.60	105520.00	0.12
Smoking	2,133	26.66	1,595	27.93	538	23.50	< 0.01
Alcohol drinking	1,560	80.48	1,155	20.25	405	17.72	0.01
Common chronic health conditions							
Hypertension	2,749	34.60	1,960	34.59	789	34.62	0.98
Arthritis	1,311	16.39	997	17.46	314	13.72	< 0.01
Cataract	813	10.43	544	9.80	269	11.98	< 0.01
Diabetes	654	8.19	442	7.75	212	9.27	0.03
Chronic lung disease	501	6.27	354	6.21	147	6.42	0.72
Angina	464	5.82	323	5.67	141	6.18	0.38
Asthma	176	2.21	115	2.03	61	2.68	0.08
Depression	37	0.46	28	0.49	9	0.39	0.56
Vegtable and fruit intake (*100g/day)	3.68	1.68	3.69	1.72	3.66	1.59	0.47
BMI	24.61	4.17	24.71	4.30	24.35	3.81	< 0.01
WHR	0.89	0.11	0.89	0.10	0.89	0.13	0.06
Physical activity level							0.56
Low level	4,801	60.01	3,407	59.66	1,394	60.90	
Moderate level	2,276	28.45	1,643	28.77	633	27.65	
High level	923	11.54	661	11.57	262	11.45	
Sedentary time (hours/day)	4.23	2.43	4.10	2.41	4.56	4.56	< 0.01
Immediate verbal recall score	5.83	1.66	5.87	1.64	5.75	1.70	< 0.01
Delayed verbal recall score	5.14	2.07	5.15	2.05	5.12	2.13	0.65
Digit span score	11.13	2.58	11.22	2.62	10.93	2.47	< 0.01
Verbal fluency score	12.53	5.36	12.34	5.25	12.99	5.60	< 0.01

*^*a*^Mean for continuous variables or number for categorical variables. ^*b*^Standard deviation (SD) for continuous variables or percentage (%) for categorical variables. ^*c*^Compared between participants who completed both cognitive assessments and those who dropped out from the second cognitive assessment.*

Table 2 compares the cognitive scores at Wave 1 and Wave 2. The mean scores in all cognitive domains significantly declined from Wave 1 to Wave 2. The mean score of immediate verbal recall decreased from 5.87 to 5.35 (*p* < 0.01), delayed verbal recall from 5.15 to 4.98 (*p* < 0.01), digit span from 11.22 to 10.00 (*p* < 0.01), and verbal fluency from 12.34 to 10.99 (*p* < 0.01).

**TABLE 2:** Comparisons of cognitive scores at Wave 1 and Wave 2.

**Cognitive domain**	**Wave 1**		**Wave 2**		**Difference**		
	***N***	**Mean (*SD*)**	***N***	**Mean (*SD*)**	***N***	**Mean (*SD*)**	***p***
Immediate verbal recall	5,705	5.87 (1.64)	5,590	5.35 (1.89)	5,584	−0.51(2.30)	<0.01
Delayed verbal recall	5,626	5.15 (2.05)	5,557	4.98 (2.15)	5,473	−0.17(2.72)	<0.01
Digit span	5,633	11.22(2.62)	5,676	10.00(3.35)	5,599	−1.19(3.81)	<0.01
Verbal fluency	5,701	12.34(5.25)	5,168	10.99(5.04)	5,158	−1.34(6.22)	<0.01

Table 3 shows the contemporaneous associations between lifestyle factors and cognitive scores from Wave 1 to Wave 2, with separate between- and within-person effects. Vegetable and fruit intake was positively associated with scores in all cognitive domains (*p* < 0.01). Per 100 g/day increase in vegetable and fruit intake was associated with an increase of 0.07 in immediate verbal recall score, 0.08 in delayed verbal recall score, 0.07 in digit span score, and 0.39 in verbal fluency score. The between-person effects were significant for all cognitive domains (*p* < 0.01), while the within-person effects were only significant for immediate verbal recall (*p* = 0.02) and verbal fluency (*p* < 0.01). For different persons at the same age, per 100 g/day increase in vegetable and fruit intake was associated with an increase of 0.10 in immediate verbal recall score, 0.14 in delayed verbal recall score, 0.09 in digit span score, and 0.37 in verbal fluency score (cross-sectional difference). For the same person from Wave 1 to Wave 2, per 100 g/day 5 year increase in vegetable and fruit intake was associated with a 5 year increase of 0.04 in immediate verbal recall score, and 0.40 in verbal fluency score (longitudinal change). Likewise, physical activity level was positively associated with scores in all cognitive domains (*p* < 0.01). Compared with subjects who had a low level of physical activity, those who had a moderate level of physical activity scored 0.17, 0.16, 0.16, and 0.99 higher in immediate verbal recall, delayed verbal recall, digit span, and verbal fluency, respectively. The between-person effects were significant for all cognitive domains (*p* < 0.01), while the within-person effects were only significant for immediate verbal recall (*p* < 0.01), delayed verbal recall (*p* < 0.01), and verbal fluency (*p* < 0.01). In contrast, BMI was negatively associated with scores in all cognitive domains (*p* < 0.01). One unit (kg/m^2^) increase in BMI was associated with a decrease of 0.02 in immediate verbal recall score, 0.03 in delayed verbal recall score, 0.03 in digit span score, and 0.05 in verbal fluency score. The between-person effects were significant for all cognitive domains (*p* < 0.01), while the within-person effects were only significant for delayed verbal recall (*p* < 0.01). WHR was negatively associated with verbal fluency score only (*p* = 0.01), and only its within-person effect was significant (*p* < 0.01). Sedentary time was negatively associated with digit span score (β = −0.03, *p* < 0.01), with a significant within-person effect (*p* < 0.01), but positively associated with verbal fluency score (β = 0.15, *p* < 0.01), with significant between- and within-person effects (*p* < 0.01). When all lifestyle factors were included in the same model (full model), all aforementioned associations remained unchanged, except the insignificant association of WHR with digit span score became significant (*p* = 0.045).

**TABLE 3:** Contemporaneous associations between lifestyle factors and cognitive scores (between- and within-person effects).

		**Immediate verbal recall**						**Delayed verbal recall**					**Digit Span**						**Verbal fluency**					
	**Estimate**	***SE***	***p*^a^**	***R*^2^**	**ICC^b^**	***p* (full model)^c^**	**Estimate**	***SE***	***p*^a^**	***R*^2^**	**ICC^b^**	***p* (full model)^c^**	**Estimate**	***SE***	***p*^a^**	***R*^2^**	**ICC^b^**	***p* (full model)^c^**	**Estimate**	***SE***	***p*^a^**	***R*^2^**	**ICC^b^**	***p* (full model)^c^**
Vegetable and fruit intake (*100g/day)	0.07	0.01	<0.01		0.12	<0.01	0.08	0.01	<0.01		0.04	<0.01	0.07	0.02	<0.01		0.07	<0.01	0.39	0.03	<0.01		0.22	<0.01
Between-person effect	0.1	0.02	<0.01	0.13			0.14	0.02	<0.01	0.13			0.09	0.03	<0.01	0.14			0.37	0.05	<0.01	0.15		
Within-person effect	0.04	0.02	0.02	0.09			0.02	0.02	0.32	0.09			0.05	0.03	0.07	0.1			0.4	0.04	<0.01	0.12		
BMI	–0.02	0	<0.01		0.21	<0.01	–0.03	0.01	<0.01		0.04	<0.01	–0.03	0.01	<0.01		0.17	<0.01	–0.05	0.01	<0.01		0.23	<0.01
Between-person effect	–0.02	0.01	<0.01	0.13			–0.02	0.01	<0.01	0.13			–0.03	0.01	<0.01	0.13			–0.06	0.02	<0.01	0.15		
Within-person effect	–0.01	0.01	0.15	0.09			–0.05	0.01	<0.01	0.09			–0.03	0.01	0.07	0.1			0	0.03	0.9	0.11		
WHR	–0.09	0.2	0.65		0.21	0.77	–0.12	0.24	0.62		0.03	0.53	0.39	0.33	0.25		0.07	0.045	–1.93	0.6	<0.01		0.24	0.02
Between-person effect	–0.12	0.27	0.67	0.09			–0.15	0.32	0.65	0.13			–0.12	0.46	0.8	0.13			–0.24	0.87	0.79	0.15		
Within-person effect	–0.06	0.3	0.84	0.12			–0.08	0.35	0.82	0.09			0.93	0.49	0.06	0.1			–3.65	0.87	<0.01	0.11		
Physical activity level	0.17	0.02	<0.01		0.19	<0.01	0.16	0.03	<0.01		0.02	<0.01	0.16	0.04	<0.01		0.09	<0.01	0.99	0.07	<0.01		0.19	<0.01
Between-person effect	0.16	0.03	<0.01	0.13			0.15	0.04	<0.01	0.13			0.28	0.06	<0.01	0.14			1.2	0.1	<0.01	0.17		
Within-person effect	0.18	0.04	<0.01	0.09			0.16	0.04	<0.01	0.09			0.03	0.06	0.6	0.1			0.8	0.1	<0.01	0.13		
Sedentary time (hours/day)	0.01	0.01	0.19		0.18	0.2	0.01	0.01	0.18		0.01	0.18	–0.03	0.01	<0.01		0.11	<0.01	0.15	0.02	<0.01		0.17	<0.01
Between-person effect	0.01	0.01	0.16	0.12			0.02	0.01	0.1	0.12			0	0.02	0.91	0.13			0.18	0.03	<0.01	0.15		
Within-person effect	0	0.01	0.66	0.09			0	0.01	0.85	0.09			–0.07	0.02	<0.01	0.1			0.12	0.03	<0.01	0.12		

*^*a*^Adjusted for age, sex, education, smoking, drinking, and self-reported common chronic health conditions including arthritis, angina, diabetes, chronic lung disease, asthma, hypertension, depression and cataract. ^*b*^Conditional intraclass correlation. ^*c*^Additionally adjusted for all other lifestyle factors of this study.*

Table 4 presents the interactions between different lifestyle factors, as well as interactions between lifestyle factor and demographic factors (age and sex). Significant interactions were observed between age and all lifestyle factors: The effects of one unit increase in vegetable and fruit intake (100 g/day), BMI, and physical activity level on digit span score increased by 0.005 (*p* = 0.046), 0.002 (*p* = 0.024), and 0.014 (*p* = 0.013), respectively, when age increased by one unit; the effects of one unit increase in BMI and sedentary time (1 h/day) on delayed verbal recall score changed by 0.001 (*p* = 0.041) and −0.003 (*p* = 0.009), when age increased by one unit. None of the interactions between sex and lifestyle factors was significant, indicating the associations of lifestyle factors with cognitive scores did not differ by sex. Among the interactions between every two lifestyle factors, only vegetable and fruit intake had significant interactions with all other lifestyle factors: For every unit increase in vegetable and fruit intake (100 g/day), the effect of BMI on delayed verbal recall score decreased by 0.009 (*p* = 0.018); the effect of WHR on immediate verbal recall score decreased by 0.337 (*p* = 0.020); the effect of physical activity level on digit span score increased by 0.054 (*p* = 0.045); and the effect of sedentary time on immediate verbal recall score and digit span score increased by 0.010 (*p* = 0.020) and 0.021 (*p* = 0.005), respectively.

**TABLE 4:** Contemporaneous associations between lifestyle factors and cognitive scores including interactions.

	**Immediate verbal recall**			**Delayed verbal recall**			**Digit span**			**Verbal fluency**		
	**Estimate**	***SE***	***p***	**Estimate**	***SE***	***p***	**Estimate**	***SE***	***p***	**Estimate**	***SE***	***p***
Vegetable and fruit intake (*100g/day)	0.196	0.164	0.234	0.369	0.197	0.061	–0.115	0.275	0.677	0.756	0.488	0.121
Vegetable and fruit intake*age	0.002	0.001	0.253	0.001	0.002	0.530	0.005	0.002	0.046	0.004	0.004	0.333
Vegetable and fruit intake*sex	0.033	0.023	0.195	0.005	0.027	0.903	0.014	0.038	0.739	0.002	0.068	0.924
BMI	–0.137	0.066	0.030	–0.171	0.079	0.030	–0.166	0.111	0.140	–0.199	0.197	0.304
BMI*age	0.001	0.001	0.142	0.001	0.001	0.041	0.002	0.001	0.024	0.003	0.002	0.103
BMI*sex	0.016	0.010	0.109	0.011	0.012	0.338	–0.019	0.016	0.266	0.014	0.029	0.631
WHR	2.561	2.249	0.255	2.982	2.690	0.267	7.088	3.768	0.059	10.490	6.653	0.115
WHR*age	–0.018	0.027	0.504	–0.039	0.033	0.238	–0.084	0.046	0.070	–0.114	0.081	0.159
WHR*sex	–0.580	0.469	0.215	–0.792	0.562	0.159	–0.357	0.784	0.649	–2.396	1.396	0.086
Physical activity level	0.145	0.400	0.717	0.522	0.480	0.277	–1.232	0.670	0.066	2.261	1.189	0.057
Physical activity level*age	0.004	0.003	0.268	–0.001	0.004	0.728	0.014	0.006	0.013	–0.012	0.010	0.237
Physical activity level*sex	0.010	0.054	0.843	0.038	0.064	0.552	–0.025	0.090	0.787	–0.208	0.160	0.202
Sedentary time (hours/day)	0.142	0.107	0.205	0.118	0.128	0.356	0.227	0.180	0.207	0.287	0.317	0.365
Sedentary time*age	–0.001	0.001	0.249	–0.003	0.001	0.009	–0.002	0.001	0.075	–0.003	0.002	0.238
Sedentary time*sex	–0.013	0.015	0.414	0.000	0.017	0.963	–0.030	0.024	0.215	–0.085	0.044	0.050
Vegetable and fruit intake*BMI	–0.002	0.003	0.549	–0.009	0.004	0.018	0.002	0.005	0.730	–0.005	0.009	0.583
Vegetable and fruit intake*WHR	–0.337	0.145	0.020	–0.232	0.173	0.180	–0.386	0.242	0.110	–0.632	0.428	0.139
Vegetable and fruit intake*physical activity level	0.016	0.016	0.336	0.021	0.019	0.279	0.054	0.027	0.045	–0.021	0.047	0.627
Vegetable and fruit intake*sedentary time	0.010	0.004	0.020	0.005	0.005	0.388	0.021	0.007	0.005	0.010	0.013	0.411
BMI*WHR	0.064	0.050	0.229	0.088	0.060	0.158	0.028	0.085	0.745	0.005	0.150	0.993
BMI*physical activity level	–0.002	0.007	0.852	–0.004	0.009	0.713	–0.001	0.012	0.930	0.011	0.021	0.571
BMI*sedentary time	0.001	0.002	0.531	0.000	0.002	0.966	–0.001	0.003	0.770	–0.010	0.006	0.087
WHR*physical activity level	–0.292	0.339	0.388	–0.455	0.407	0.254	0.312	0.567	0.578	–0.602	1.005	0.549
WHR*sedentary time	–0.128	0.099	0.194	0.016	0.118	0.881	–0.149	0.166	0.355	0.438	0.293	0.135
Physical activity level*sedentary time	0.000	0.011	0.990	0.014	0.013	0.259	0.019	0.018	0.287	–0.015	0.032	0.657

*Adjusted for age, sex, education, smoking, drinking, and self-reported common chronic health conditions including arthritis, angina, diabetes, chronic lung disease, asthma, hypertension, depression and cataract.*

To allow for a time lag between lifestyle factors and cognitive changes, we also examined the associations between baseline lifestyle factors and longitudinal cognitive scores (Supplementary Table 1), as well as the interactions between different baseline lifestyle factors and the interactions between baseline lifestyle factors and demographic factors (age and sex) (Supplementary Table 2). These results differ from the contemporaneous associations in Tables 3, 4, which supports our assumption that the fluctuations in lifestyle behavior over time affect the predictive value of lifestyle behavior for future cognitive performance.

## Discussion

This large-scale prospective cohort study has been one of the first attempts to examine the contemporaneous associations of a broad spectrum of time-varying modifiable lifestyle factors with cognitive performance in older Chinese adults. Overall, our study suggests that better management of multiple lifestyle factors may protect against cognitive decline in later life. Specifically, we have found that higher vegetable and fruit consumption and a higher level of physical activity were positively associated with all cognitive domains, whereas BMI was negatively associated with all cognitive domains. Sedentary time was negatively associated with digit span score but positively associated with verbal fluency score. The between-person effects seem to be more dominant than within-person effects.

Our results seem to be consistent with previous research which has shown that the adherence to overall healthier lifestyles are associated with better cognitive function in adulthood (Lövdén et al., 2013; Klimova et al., 2017; Kivipelto et al., 2018; Bott et al., 2019; Mintzer et al., 2019). The mechanisms that underlie the relationship between lifestyle factors and cognition are not yet fully understood. Several hypotheses have been proposed that highlight the involvement of vascular, inflammatory, oxidative stress, neurotoxic and psychosocial processes (Kivipelto et al., 2018). Much more extensive research is available in the area of diet and physical activity compared to other lifestyle factors. Overall, fruits and vegetables were the most common dietary elements associated with better cognitive function. This study confirms that higher vegetable and fruit consumption and physical activity are protective against cognitive decline in older adults. Vegetables and fruits are rich in antioxidant vitamins and nutrients, compounds that are considered important for the protection against oxidative stress and inflammation, which, in turn, have been shown to play a role in the early pathophysiology of cognitive decline (Hajjar et al., 2018; Gehlich et al., 2019). Moreover, vegetables and fruits might affects the composition of the gut microbiota and stimulate a positive modulation of the gut–brain axis, which might be another mechanistic pathway to impact cognitive health (Pistollato et al., 2016). A previous prospective study of 3,718 elderly participants reported that the decrease in cognitive decline over 6 years of follow-up for people who consumed greater than two vegetable servings per day was equivalent to about 5 years of younger age on cognitive testing (Morris et al., 2006). Similarly, strong observational data have identified physical activity as a potent lifestyle factor that plays a critical role in alleviating age-related cognitive decline across the life span (Prakash et al., 2015; Engeroff et al., 2018). It is hypothesized that the neural and vascular adaptations to physical activity improve cognition through promotion of neurogenesis, angiogenesis, synaptic plasticity, decreased proinflammatory processes and reduced cellular damage due to oxidative stress (Northey et al., 2018). A meta-analysis of cohort studies concluded that as compared with adults not engaged in physical activity, those with a high level of physical activity showed 38% less decline in cognitive performance during a 1–12 years of follow-up, and even those with a low-to-moderate level of physical activity also showed 35% less decline (Sofi et al., 2011). A more recent meta-analysis examining the effects of exercise interventions on cognitive outcomes in adults aged 50 and older reported an overall small-to-moderate, but significant, effect size for all outcomes of cognition evaluated (i.e., attention, executive function, memory, and working memory), which is in agreement with our findings (Northey et al., 2018).

On the other hand, the existing literature on the relationship between obesity and cognition has yielded contradictory and age dependent findings. While obesity in midlife appears to be detrimental to cognitive decline, obesity in the old (over 65 years) has been reported to be detrimental, neutral or even protective, highlighting an “obesity paradox” (Sellbom and Gunstad, 2012; Bischof and Park, 2015; Monda et al., 2017). These paradoxical findings may be explained by a survival effect in elderly samples. Namely, some middle-aged obese adults experience mortality, leaving a healthier sample of obese adults in old age (Smith et al., 2011; Bischof and Park, 2015). Furthermore, some researchers have argued that BMI, which is the most widely used indicator of obesity in studies with respect to cognitive decline, is not a good measure of body composition in the old (Bischof and Park, 2015). Lean body mass decreases, while adipose tissue increases without weight gain during aging (Monda et al., 2017). This distribution of lean and fat tissue masses may not be captured by BMI when evaluating adiposity in the elderly (Monda et al., 2017). Central adiposity (i.e., waist circumference or waist-to-hip ratio), on the other hand, has been proposed as a better adiposity marker in old age (Bischof and Park, 2015; Monda et al., 2017). On the question of the “obesity paradox,” we further investigated the relation of obesity and cognitive performance stratified by baseline age (≤65 and >65) (Supplementary Table 3). Our stratified analysis showed that the negative associations of BMI with all cognitive domains were only significantly in people up to 65 years old (*p* < 0.01), but not in people older than 65 (*p* > 0.05). The positive association of WHR with digit span was only significant in people up to 65 years old in the full model (*p* = 0.01), while the negative association of WHR with verbal fluency was only significant in people older than 65 (*p* = 0.01). The results of our stratified analysis seems to reflect the aforementioned “obesity paradox” phenomenon and differential findings of BMI and WHR with respect to cognitive decline. Overall there is currently not enough evidence to conclude a reliable association between obesity and cognitive decline in older adults. More research is needed to understand the complex nature of the effects of obesity on cognition.

Our study found that the between-person associations were more dominant than the within-person associations for some lifestyle factors, particularly for BMI, which might be attributed to the relatively short follow-up period and the extent of within-person changes in lifestyle factors. First, our study accessed the within-subject effects over 5 years, however, the within-subject effects might need a longer follow-up period to manifest. Second, small within-person changes in lifestyle factors might not result in significant effects on cognitive functioning. The extent of within-person changes varied widely among different lifestyle factors in our participants. Within a 5-year follow-up period, only 30.5% of the participants changed BMI group, 40.1% changed WHR group, 51.2% changed physical activity level, 70.0% had more than 20% change in vegetable and fruit intake, and 80.9% had more than 20% change in sedentary time. The stability in BMI seems to explain the insignificant within-person associations for all cognitive domains. More significant within-person associations were observed for other lifestyle factors with greater within-person changes, such as WHR, physical activity level, vegetable and fruit intake, and sedentary time. Nevertheless, people should engage in a healthy lifestyle, so that it could have an impact on cognitive function in later life.

Our study found many significant interactions between different lifestyle factors, suggesting potential interactive effects among lifestyle factors on cognition. The etiology of cognitive decline and dementia is multifactorial and has many concurrent modifiable risk factors and protective factors (Kivipelto et al., 2018). To date, lifestyle intervention studies that have aimed to prevent cognitive decline and dementia have mainly been single-domain trials, in which only single behavior was targeted (Lövdén et al., 2013; Kivipelto et al., 2018). Such single-domain approach did not consider the possible interactive effects between different lifestyle factors, and is unable to provide overall and integrated recommendations for how to slow down cognitive decline and prevent dementia in the general population. Consequently, studies targeting many lifestyle factors simultaneously may help understand the combined effects of lifestyle factors on cognitive decline, and offer the best approach to an optimal preventive effect. Evidence from the first large multi-domain lifestyle intervention trial (FINGER) has provided some support for the conceptual premise that multi-domain lifestyle intervention is an effective strategy (Ngandu et al., 2015). However, two other large multi-domain lifestyle intervention trials (MAPT and PreDIVA) have found negative results (Moll van Charante et al., 2016; Andrieu et al., 2017). In addition to these three large multi-domain trials, several small-scale trials have yielded conflicting findings too. Owing to the limited sample size and duration of these trials, the efficacy of multi-domain lifestyle intervention for cognitive decline remains inconclusive. Further research exploring the combined effects of a broad range of lifestyle factors on cognitive decline with a long follow-up period is warranted.

Strengths of this study include the longitudinal cohort design, a large population-based sample, and a long period of follow-up. Further strength includes repeated measures of synchronous lifestyle factors and cognitive performances and the evaluation of contemporaneous associations between them, which is rarely available in prior research. Most notably, this study examined a broad range of lifestyle factors on cognitive decline simultaneously, and therefore could provide overall and integrated recommendations on prevention against cognitive decline. A limitation of this study is the potential attrition bias due to differential loss to follow-up with respect to the lifestyle variables and cognitive outcomes. Another limitation is that the cognitive measures used in this study do not have immediate clinical relevance in terms of risk for mild cognitive impairment (MCI) and dementia. Future research that uses MCI as a measure of cognitive functioning would have more important implications at a population level. Finally, our sample is only representative of the older adults in Shanghai, not all of China.

In summary, the present study has been one of the first attempts to comprehensively determine the contemporaneous associations of a broad spectrum of time-varying modifiable lifestyle factors with age-related cognitive decline in older adults. Our findings suggest that better management of multiple lifestyle factors may protect against cognitive decline in later life. Higher vegetable and fruit consumption and physical activity are protective, whereas obesity is detrimental to cognitive decline in older adults. This study underpins the development of multi-domain lifestyle recommendations to promote healthy cognitive aging. As lifestyle behavior is a self-regulating and relatively easy target to prevent cognitive decline, the public health relevance of such a non-pharmacological approach is an important consideration. In the context of the fast growing aging population and dementia patients, even modest positive effects on cognitive function related to greater adherence to an overall healthy lifestyle could yield significant public health benefits if they can be cost-effectively delivered on a large scale. Especially in low- and middle-income countries with limited health-care resources, lifestyle intervention might represent one cost-effective general prevention strategy. More longitudinal randomized controlled trials targeting multi-domain lifestyle intervention are needed to determine the effectiveness of multi-domain lifestyle intervention and inform future lifestyle recommendations for older adults to prevent cognitive decline.

## Data Availability Statement

The raw data supporting the conclusions of this article will be made available by the authors, without undue reservation.

## Ethics Statement

The studies involving human participants were reviewed and approved by the Shanghai Center for Disease Control and Prevention Ethical Review Committee. The patients/participants provided their written informed consent to participate in this study.

## Author Contributions

ZH contributed to the design of the study, data collection and analysis, and preparation of the manuscript. YG, YR, SS, YS, and FW contributed to the design of the study, data collection, and preparation of the manuscript. TL, JY, JL, LH, and SW contributed to the data collection and processing. All authors contributed to the article and approved the submitted version.

## Conflict of Interest

The authors declare that the research was conducted in the absence of any commercial or financial relationships that could be construed as a potential conflict of interest.
