# Supplementary material for: Associations of Lifestyle Factors With Cognition in Community-Dwelling Adults Aged 50 and Older: A Longitudinal Cohort Study
Source: Front Aging Neurosci. 2020 Nov 9;12:601487. doi: 10.3389/fnagi.2020.601487 (PMC7680821; doi:10.3389/fnagi.2020.601487)
Supplement: Supplementary file 1 [file Table_1.DOCX]

Supplementary Material

**Supplementary Table 1.** Associations between baseline lifestyle factors and cognitive scores

^a^ Adjusted for age, sex, education, smoking, drinking, and self-reported common chronic health conditions including arthritis, angina, diabetes, chronic lung disease, asthma, hypertension, depression and cataract.

^b^ Additionally adjusted for all other lifestyle factors of this study.

**Supplementary Table 2.** Associations between baseline lifestyle factors and cognitive scores including interactions

Adjusted for age, sex, education, smoking, drinking, and self-reported common chronic health conditions including arthritis, angina, diabetes, chronic lung disease, asthma, hypertension, depression and cataract.

**Supplementary Table 3.** Associations between obesity and cognitive scores stratified by baseline age (<=65 and >65)

^a^ Adjusted for age, sex, education, smoking, drinking, and self-reported common chronic health conditions including arthritis, angina, diabetes, chronic lung disease, asthma, hypertension, depression and cataract.

^b^ Additionally adjusted for all other lifestyle factors of this study.
